# Evaluation of tendon healing using fibroblast like synoviocytes in rabbits: A biomechanical study

**Published:** 2016-03-15

**Authors:** Mahboobeh Azad-Tirgan, Farshid Sarrafzadeh-Rezaei, Hassan Malekinejad, Rahim Hobbenaghi, Behnam Heshmatian

**Affiliations:** 1 *Department of Surgery and Diagnostic Imaging, Faculty of Veterinary Medicine, Urmia University, Urmia, Iran; *; 2 *Department of Basic Sciences, Faculty of Veterinary Medicine, Urmia University, Urmia, Iran; *; 3 *Department of Pathobiology, Faculty of Veterinary Medicine, Urmia University, Urmia, Iran;*; 4 *Neurophysiology Research Center, Urmia University of Medical Sciences, Urmia, Iran.*

**Keywords:** Biomechanical properties, Fibroblast like synoviocytes, Rabbit, Tendon

## Abstract

Tendon never restores the complete biological and mechanical properties after healing. Several techniques are available for tissue-engineered biological augmentation for tendon healing like stem cells. Recently, synovium has been investigated as a source of cells for tissue engineering. In the present study, we investigated potentials of fibroblast like synoviocytes (FLSs) in tendon healing. Sixteen rabbits were divided randomly into control and treatment groups. One rabbit was used as a donor of synovial membrane (synovium). The injury model was unilateral complete transection through the middle one third of deep digital flexor tendon (DDFT). Subsequently, the tendon stumps were sutured with 3/0 nylon. In treatment group, 0.1 mL phosphate-buffered saline (PBS) solution containing 1 × 10^6^ nucleated cells of FLSs was injected intratendinously at both tendon stumps just next to incision line. In control group, 0.1 mL PBS without FLSs was used for intratendinous injection. Model animals were euthanized at eight weeks, DDFTs were harvested and prepared for biomechanical study. Results of study showed that, there was no significant differences in biomechanical parameters values between FLSs treated and control groups. In conclusion, intratendinous injection of FLSs did not improve biomechanical properties during eight weeks in rabbit.

## Introduction

The principal function of tendon is to transfer forces between muscles and related bones.^1^ Tendon injuries are among the most common health problems affecting the adult population^2^ and result in important morbidities.^3^ Tendon injuries can be acute or chronic and caused by intrinsic or extrinsic ingredient, either alone or in combination.^4^ Few information about these tissues in comparison to other musculoskeletal tissues such as bone, cartilage and muscle is available.^1^

Researchers believed that the structure of the synovial membrane in the tendon sheath is similar to that in the joint, but there have been a few morphological studies about tendon sheath. The inner surface of the tendon sheath like the joints, covered with a cell-rich intimal layer.^5^ Recent studies have shown that synovial cells in the synovial sheath have the potential to migrate to the injury site and accelerate connective tissue healing.^6^ Tendon has poor healing capacity and often requires medical or surgical intervention following injuries,^1^ because the biomechanical properties of the injured tendon remain suboptimal due to the lack of proper structural organization and poor matrix formation. Subsequently, a larger amount of fibrous tissue is needed to compensate for this biomechanical insufficiency, which results in a thickened but stiffened tendon and increased local adhesions.^7^

Several new techniques like use of growth factors, gene therapy and mesenchymal stem cells (MSCs) are available for tissue-engineered biological augmentation in tendon healing.^3^ Tissue engineering is an emerging technology that offers a novel approach for treating tendon injuries. The most common tissue-engineering principles are (i) the use of healthy multipotent cells that are non-immunogenic, easy to isolate and highly responsive to distinct environmental cues; (ii) the development of carrier scaffolds that provide short-term biomechanical stability of the transplant as well as a template for spatial growth of the regenerating tissue; and (iii) the delivery of growth factors that drive the process of cell differentiation and maturation.^8^ Recently, synovium has been investigated as a source of cells for meniscal ﬁbrocartilage engineering because of its high potential in regeneration following subtotal synovectomy, and its spontaneous chondrogenic behavior observed during the process of synovial chondromatosis.^9^

Experimental induction of synovial MSCs chondro-genesis has been well-demonstrated in the *in vitro* setting, thus making the cell source promising for cartilage-based tissue engineering strategies.^9^ The researchers reported effects of synovium-derived fibroblast trans-plantation to the tendon defect^10^ and tendon autograft for anterior cruciate ligament reconstruction.^11^^,^^12^ MSCs derived from synovium have higher proliferation and differentiation potentials than the other MSCs.^3^

The present study was conducted to evaluate the hypothesis that whether intratendinous injection of fibroblast like synoviocytes (FLSs) would improve the biomechanical properties of experimentally induced tendon injury in rabbits. 

## Materials and Methods

All procedures were carried out in accordance with the guidelines of the Ethics Committee and Urmia University Research Council approved all experiments. 

Sixteen skeletally mature female rabbits (2.5 to 3.0 kg) were used. The animals were divided randomly into the two groups of eight rabbits and one rabbit was used as a donor of synovium.

**Fig. 1:**
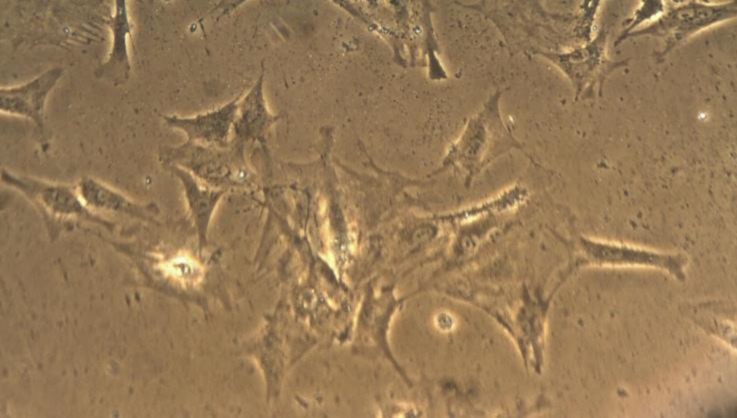
Appearance of FLSs (passage 4) growing in cell culture. Note the elongated appearance that characterized by branched cytoplasmic processes (400×).

**Preparation of FLSs** **.** Synovial membrane was harvested from the suprapatellar bursa of the knee through a medial parapatellar approach from donor rabbits.^10^ Following the removal of fats by sharp dissection, the synovium were minced into 2 to 3 mm^2^ pieces and treated for 4 hr with 4 mg mL^-1^ of collagenase type I (C6885, Sigma-Aldrich, St Louis, USA), in Dulbecco’s modified Eagle’s medium (DMEM; Sigma Co., St. Louis, USA) at 37 ˚C in 5% CO_2_. After digestion, the synoviocytes and chondrocytes suspensions were filtered (cell strainer porosity 70 µm), centrifuged for 5 min at 500 *g*, and then washed three times in their respective culture medium. The cells were counted and their viability was quantified by 0.4% trypan blue exclusion in phosphate buffered saline solution (PBS) before further culture. Dissociated cells were then centrifuged for 5 min at 500 *g*, re-suspended in DMEM supplemented with 10% fetal calf serum, 2 mM L-glutamine, penicillin (100 units mL^-1^) and streptomycin (100 mg mL^-1^) and then plated in 75-cm^2^ flasks. Cultures were kept at 37 ˚C in 5% CO_2_, and the medium was replaced every three days. When cells approached 90% confluence, they were passed by diluting 1:3 with fresh medium and were re-cultured until used. After three to four passages, the proliferating FLSs were the dominant cell type (Fig. 1). The number of cells was counted by Neubauer slide. 

**Surgical procedure. **Rabbits were anesthetized under intramuscular anesthesia (5 mg kg^-1 ^xylazine HCl and 40 mg kg^-1^ ketamine HCl; Alfasan, Woerden, The Netherlands). One hind limb of each rabbit was selected randomly and prepared for aseptic surgery. Skin was incised longitudinally on the plantar aspect of middle third of the metatarsus over the flexor tendons. The sub-cutaneous tissues were dissected and the deep digital flexor tendon (DDFT) was exposed. The injury model was a sharp complete transection through the central one third of the tendon. Subsequently, the tendon stumps were sutured with 3/0 monofilament nylon (Ethicon Inc., Somerville, USA) in modified Kessler pattern. Then, 0.1 mL PBS solution containing 1 × 10^6^ nucleated cells of FLSs was injected intratendinously at both tendon stumps just next to incision line in FLSs treated group. Rabbits in control group underwent the same surgical procedures, except that they just received the equal volume of PBS solution for intratendinous injection. The skin was closed with simple interrupted pattern with 3/0 nylon suture (Ethicon Inc.). A below-stifle fiberglass cast was applied after surgery and immobilization was continued for two weeks. No anti-biotics were used during the study. 

**Biomechanical testing. **Eight weeks after surgery, all rabbits were euthanized with intravenous injection of thiopental sodium overdose (50 mg kg^-1^, Sandoz, Kundl, Austria) and the surgical sites were reopened. Tendons were harvested by proximal and distal transverse incisions approximately 2 cm away from the repair site. Additionally, 16 intact (un-operated) tendons from contralateral limbs were harvested and assigned for biomechanical testing. All samples were wrapped into sterile moisturized gauze with normal saline and stored at – 20 ˚C until tensile testing. Before testing, tendons were removed from the freezer, thawed for 2 hr at room temperature. Sutures were removed from the operated tendons in both groups, before tensile testing. All tests were performed at room temperature. Tendons were submitted to the traction biomechanical testing machine (Series Z010; Zwick/Roell, Ulm Germany). The two ends of the tendons were clamped in the serrated jaws of the machine. In order to prevent tendon slippage during tensile testing, 360 grit sandpaper was attached to the ends of each specimen for enhanced clamping. The distance between the clamping jaws was set to 30 mm. The upper clamp was attached to a 500 N load cell and its displacement was controlled with a computer. Dynamic testing took place under axial tension with a constant speed of 50 mm min^-1^. The biomechanical testing consisted of a single-cycle load-to failure. The force and elongation of the tendon were continuously recorded until the tendon failed. For each test, the force-elongation curve was plotted and the following biomechanical parameters were obtained: ultimate (N), energy absorption (N mm), stiffness (N mm^-1^), yield load (N), stress (N mm^-2^) and strain (mm mm^-1^). The ultimate load was defined as the maximum load force measured in the tendon during the failure test. Energy absorption values were measured by calculating the area under the force-elongation curve, up to the point of maximum force. Stiffness was determined as the maximum gradient in the linear region of the force-elongation curve. Yield load was defined as the stress at which an elastic material under increasing stress ceases to behave elastically. Stress is force per unit area and strain is defined as deformation of a solid due to stress. The parameters were calculated as percentage of the values of the healing tendon as compared to the un-operated contralateral limb of the same animal rather than using absolute testing values, in order to minimize the biological variation of tensile stress among individuals.^11^

**Statistical Analysis. **Statistical analysis of data was carried out using *t*-test in Minitab (Version 16; Minitab Inc., State College, USA). A *p*-value less than 0.05 was considered significant.

## Results

**Fig. 2:**
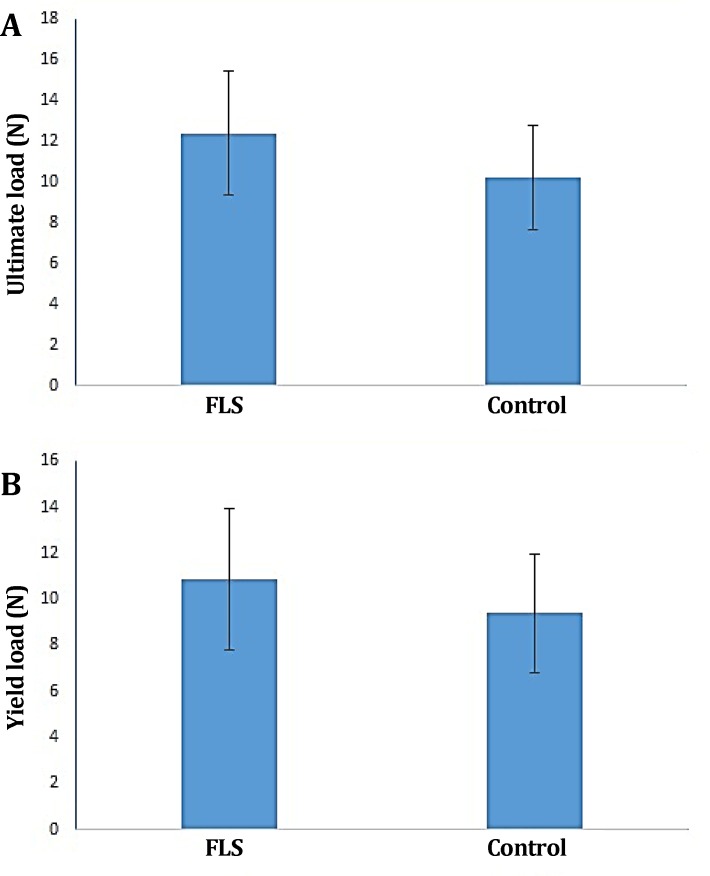
**A)** Ultimate load and **B) **Yield load of FLS-treated group compared to the control tendons. No significant difference observed between treatment and control groups

**Fig. 3:**
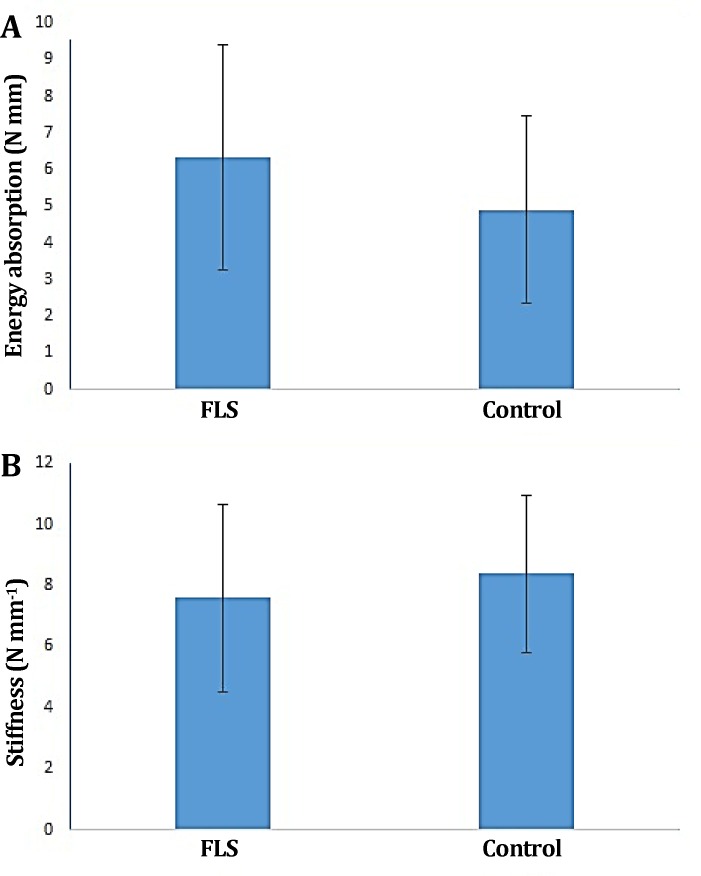
**.** **A)** Energy absorption and **B)** Stiffness of FLS-treated group compared to the control tendons. No significant difference observed between treatment and control groups

**Fig. 4:**
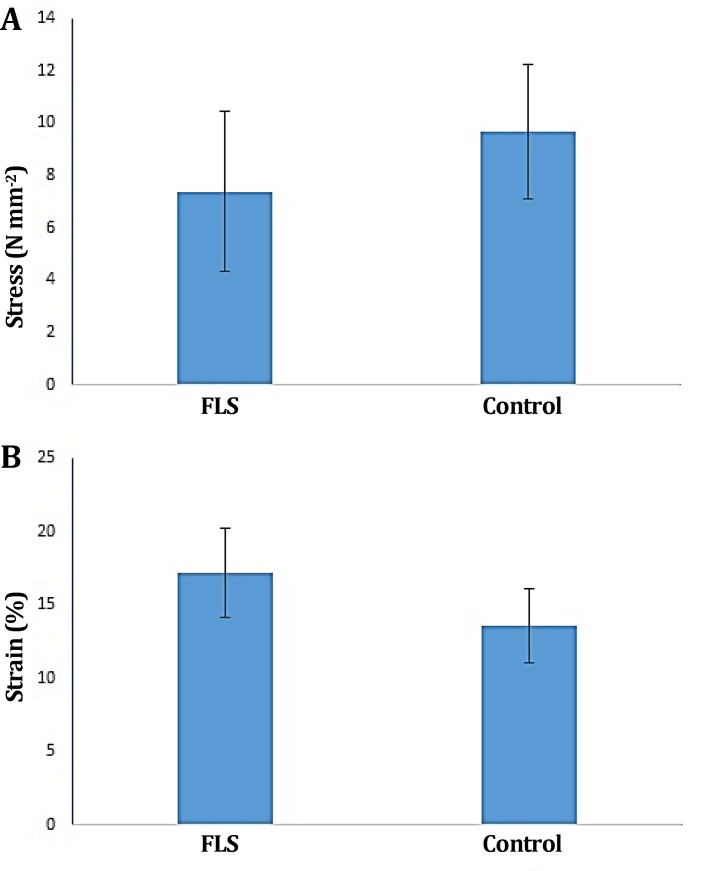
**A)** Stress and **B)** Strain of FLS-treated group compared to the control tendons. No significant difference observed between treatment and control groups

There was no evidence of faulty union and/or local or systemic complications. There were no significant differences in the ultimate load, energy absorption, stiffness, yield load, stress and strain between treatment and control groups (*p *> 0.05), (Figs. 2, 3 and 4). 

## Discussion

The purpose of this study was to clarify the effect of cell therapy with FLSs on the biomechanical properties of DDFT, following experimental tenotomy followed by tenorrhaphy. In the present study, we used FLSs without any growth factors, because other researchers demonstrated that this type of cell plays an important role in healing of meniscal regeneration under the effects of growth factors^9^ and in cruciate ligament reconstruction activated by growth factors, ^12^ and we aimed to clarify the effects of cell in healing tendon *per se*. 

Our study demonstrated that there was no significant difference between treatment and control groups. Using a rabbit patellar tendon defect model, Awad *et al*. found that implantation of a mesenchymal stem cells collagen gel composite to the defect significantly increased the tangent modulus and the tensile strength of the regenerated tissue in comparison with implantation of only collagen gel at 12 and 26 weeks, although they did not find significant effects of the implantation at six weeks.^13^ Our evaluation was performed at eight weeks after the operation and it can be a limitation of our study. Also, Okamoto *et al*. showed that transplantation of autologous synovium-derived fibro-blasts into the tendon defect, reduced the biomechanical properties of the regenerated fibrous tissue at six weeks. In their study, biomechanical properties of the regenerated tissues (tangent modulus, tensile strength) in FLSs treated group were significantly less than that of control group but they did not obtain significant differences in the strain at failure among groups. However, the *ex vivo* supplementation of transforming growth factor beta-1 (TGF-β1) into the medium significantly decreased mechanical deterioration of the fibrous tissue regenerated in the tendon defect after transplantation of cultured autologous synovium-derived fibroblasts. Their study suggested that the effect of fibroblast transplantation is significantly affected by supplement for cell culture.^10^ In addition, Kondo *et al*. demonstrated that local application of synovium-derived fibroblasts significantly accelerates extrinsic fibroblast infiltration into the tendon graft at 12 weeks after anterior cruciate ligament reconstruction (ACL reconstruction), but the application results in significant reduction of the structural properties of the graft. The reduction is considered to be not only due to the flaw effect of cell infiltration but also due to biological activities of the infiltrating cells with regard to collagen synthesis and destruction.^14^ Tohyama *et al*. reported that the rapid infiltration of extrinsic cells into the patellar tendon after the in situ freeze-thaw treatment induces mechanical deterioration of the patellar tendon.^15^ Kraus *et al*. showed that MSCs have only partially positive effects on tendon remodeling in the initial stages; however, in later stages, stem cells have potentially negative effects on bio-mechanical results and the additional expression of basic fibroblast growth factor in stem cells has negligible effects on tendon remodeling.^16^ Also, Young *et al*. demonstrated that implanted synovial MSCs improve early remodeling of the tendon-bone healing at 1 and 2 weeks histologically.^17^ However, the effect was not observed at 4 weeks but Lei Sun *et al*. found that joint synovial fluid appears to have an inhibitory effect on tendon-to-bone healing in rabbits at an early stage.^18^

It is widely accepted that use of synovium-based cells for cartilage engineering requires induction of the cartilage phenotype through cytokine or hormone stimulation.^9^ Fox *et al*. demonstrated FLSs-seeded scaffold does not exhibit the capacity to integrate into avascular meniscal tissue *in vitro*, however, their data suggested that FLSs may constitutively signal for production of type I collagen, and can be induced to signal for collagen II and aggrecan which may prove favorable for *in vitro* fibrocartilage tissue engineering under appropriate conditions. Furthermore, under the effects of TGF-beta and insulin-like growth factors (IGF1), FLSs express more alpha smooth muscle actin, which may be an important cellular response to mediate avascular meniscal healing.^9^ These studies implied that cell culture condition is one of the important factors that lead potential cell-based therapy for tendon and ligament regeneration to success.^10^ In an experimental study on rabbits, biomechanical testing showed improved modulus in the bone marrow MSCs group as compared with the control group at three weeks but not at six and 12 weeks.^19^ In FLS-treated group, we transplanted only cells without any scaffold in order to clarify pure effects of cell transplantation as distinguished from effects of carriers, but Dunn *et al*. reported that the collagen scaffold promotes fibroblast attachment and increases the rate of cell proliferation and neocollagen synthesis *in vitro* and they showed that fibroblast function depends on the tissue culture substrate and the origin of the fibroblasts.^20^ Synovium-derived cells had the greatest potential on both proliferation and chondrogenesis, indicating their usefulness for cartilage study in a rat model.^21^ Among the MSCs derived from mesenchymal tissues, Yoshimura *et al*. have found that synovium-derived cells have the highest colony-forming potential and proliferation rate in culture. This indicates the usefulness of synovium-derived cells for cartilage study in a rat model.^21^ Results of another study have shown that the biomechanical benefit of stem cells can be seen if the study is followed up over a longer period.^22^ We believe that FLSs need adjuncts to be most effective. More studies are required to understand the healing outcome and fate of these cells when implanted in different clinical models. Because of poor study designs and/or missing long-term results about FLSs, the benefits and transferability to clinical use are often limited. Further studies are needed to determine if cell-based strategies need to be combined with growth and differentiation factors to be effective.

In conclusion, results of present study demonstrated that fibroblast like synoviocytes do not show any beneficial effects on biomechanical properties of the deep digital flexor tendon of rabbit tendon at eight weeks.
